# ﻿Species delimitation in the genus *Klebsormidium* (Klebsormidiophyceae, Charophyta), including description of *Klebsormidium mirabile* sp. nov. with high content of polyunsaturated fatty acids

**DOI:** 10.3897/phytokeys.266.158514

**Published:** 2025-11-07

**Authors:** Yulduzkhon Tukhtaboeva, Elena Krivina, Vera Redkina, Aleksey Portnov, Maria Sinetova, Anna Temraleeva

**Affiliations:** 1 Kimyo International University of Tashkent, 160100 Namangan, Uzbekistan; 2 All-Russian Collection of Microorganisms (VKM), G.K. Skryabin Institute of Biochemistry and Physiology of Microorganisms, Pushchino Scientific Center for Biological Research, Russian Academy of Sciences, 142290 Pushchino, Russia; 3 Institute of Physico-Chemical and Biological Problems in Soil Science, Pushchino Scientific Center for Biological Research of the Russian Academy of Sciences, Russian Academy of Sciences, 142290 Pushchino, Russia; 4 К.А. Timiryazev Institute of Plant Physiology Russian Academy of Sciences, 127276 Moscow, Russia

**Keywords:** Algae, fatty acid profile, integrative taxonomy, machine learning, morphology, new species, phylogeny, soil

## Abstract

The genus *Klebsormidium* is a cosmopolitan filamentous alga that belongs to the phylum Charophyta and inhabits a wide range of environments, primarily freshwater, soil, and aerial habitats. Its basal position on the Streptophyta phylogenetic tree, widespread distribution and significant functional role make the genus *Klebsormidium* an important object for phycological research. To advance the molecular phylogeny of *Klebsormidium*, we conducted the first comparative evaluation of six species delimitation methods – ASAP (Assemble Species by Automatic Partitioning), LocMin (Local Minima), KoT (K over Theta), GMYC (Generalized mixed Yule-coalescent), mlPTP (Poisson Tree Process with Maximum Likelihood), and bPTP (Poisson Tree Process with Bayesian inference) – using *rbc*L and concatenated ITS2–*rbc*L fragments. The accuracy of these methods was further validated against molecular, morphological, and ecological data. According to an integrative approach, *Klebsormidium mirabile***sp. nov.** was proposed as a new charophyte species from soil of the Fergana Valley, Uzbekistan. In addition, a study on the fatty acid composition showed that the authentic strain VKM Al-436 of this new species of *Klebsormidium* is a potential source of polyunsaturated fatty acids, including linoleic, α-linolenic, and hexadecatrienoic acids.

## ﻿Introduction

The discovery of new species, at a time when many are in a rapid decline, plays a pivotal role not only for fundamental, but also applied research, because biodiversity ensures the health of ecosystems and therefore human societies on the whole (Diaz et al. 2006). Species are the basic taxonomic units used to study evolutionary biology and biodiversity, and also assigned a key category in nature conservation and the development of environmental policy. Additionally, some scientists state that the species is the most ‘real’ unit of taxonomy, with higher taxa representing historical entities (Eldredge and Cracraft 1980; Wiley 1981).

Species delimitation has traditionally been based on morphological features for the vast majority of eukaryotic algae. However, in recent decades, phycologists have included obligatory molecular data in the descriptions of new taxa and their taxonomic revisions, leading to significant changes in established taxonomic classifications (Leliaert et al. 2014). Several methods have been suggested for distinguishing between species of algae. The classical approach uses a pairwise divergence of variable molecular markers, for example a 2–10% divergence in ITS2, as recommended by Hoshina et al. (2010, 2021), as a threshold for species separation. However, the rates of nucleotide substitutions can vary among different evolutionary lineages, so do the levels of variation within and between species, which makes it difficult to establish a universal threshold (Loren et al. 2018). Another widely used method for species delimitation is based on searching for at least one CBC in the conserved regions of the ITS2 secondary structure (Coleman 2000, 2009). This approach also has its limitations (Müller et al. 2007; Caisová et al. 2011). Despite the popularity of these methods, accurate species identification remains a challenging task that has led to the development of more sophisticated, machine learning-based methods (De Queiroz 2007; Fujita et al. 2012). Nevertheless, when comparing any DNA-based methods with traditional phenotypic approaches, there are sometimes discrepancies. In this context, nowadays, the polyphasic or integrative approach is most preferable. It is based on determination of whether a group of individuals belongs to the same or different species in compliance with their genetic, morphological, ecological, biochemical, or other characteristics. Furthermore, such an approach can combine multiple evidence sets, as well as alternative methods for delimiting species (for example, threshold-, character-, tree-, and coalescence-based methods).

The cosmopolitan filamentous algae of the genus *Klebsormidium* P.C.Silva, Mattox & W.H.Blackwell belongs to the phylum Charophyta and inhabits a wide range of environments, primarily freshwater, soil, and aerial habitats (Samolov et al. 2019). As of today, this genus includes 25 taxonomically accepted species (Guiry and Guiry 2025), some of them described recently (Novis 2006; Samolov et al. 2019). The attention to this genus has not faded due to its basal position on the Streptophyta phylogenetic tree, the study of which is essential for understanding the origins and the evolutionary trends of land plants (Sluiman et al. 2008; Hori et al. 2014). Moreover, the widespread distribution and significant functional role of *Klebsormidium* as its ability to form biological soil crusts and aerial biofilms that provide microbial hotspots in terrestrial ecosystems, make it an important subject for phycological research. *Klebsormidium* representatives were able to spread so widely due to their ability to survive harsh environmental conditions including UV radiation, high and low intensity light, temperature fluctuations, and extended air drying periods (Karsten et al. 2016; Stoyneva-Gärtner et al. 2019). Many *Klebsormidium* strains were reported as rich in polyunsaturated fatty acids (PUFAs), especially linoleic and α-linolenic acids (Buse et al. 2013; Liu et al. 2016; Qiu et al. 2020; Santunione et al. 2024). These FAs are usually the main components of microalgal chloroplast membranes and are responsible for the chloroplast structure and maintaining normal photosynthetic functions, especially under stress conditions (Guschina and Harwood 2006; Los et al. 2013).

In the present study, we compared the levels of efficacy for six different species delimitation methods (ASAP, LocMin, KoT, GMYC, mlPTP, and bPTP), and verified the accuracy of the results of these methods with other molecular, morphological and ecological data for *Klebsormidium* species. We also described *K. mirabile* sp. nov., isolated from soil in the Fergana Valley (Uzbekistan), and studied the fatty acid profile of its authentic strain VKM Al-436.

## ﻿Materials and methods

### ﻿Study site and sampling

The study area was located in the northern part of the Fergana Valley, specifically in the Namangan region of Uzbekistan. The soil samples were collected in the Chust district of Namangan region, at the Chust-Pop Hills with an elevation of 496 m above sea level (40°52'37.14"N, 70°59'44.76"E). The soils were classified as poorly developed gray-brown soils (Regosols Ochric) that were formed under the influence of erosion and salinization. Soil samples were collected in April 2022 from the upper humus layer at a depth of 5 cm. The samples were collected in triplicate (10 g each) using a metal spatula that was flame-sterilized with 96% ethanol between each sampling. The samples were placed in sterile containers and transported to the laboratory for analysis.

### ﻿Isolation and cultivation of algal strains

Mixed algal cultures were obtained using soil-water enrichment and plate culture, as well as inoculating Bristol and BG-11 solid and liquid media with soil suspensions. Monocultures were isolated using the streak plate method and by isolating individual colonies with a Pasteur pipette. An axenic culture was established by repeatedly picking visually pure filaments and transferring them to fresh agar medium. Culture purity was confirmed by the absence of bacterial growth after inoculation onto LB agar. The studied strain was grown in a climate chamber under standard conditions (23–25 °C, 60–75 μmol quanta m^-2^ s^-1^, 12-hour light period) on BG-11 agar medium with nitrogen (pH 7.0, 1.4% agar) and then deposited at the All-Russian Collection of Microorganisms (VKM), with the number VKM Al-436. To maintain the axenic culture, the same medium was used, with the addition of 1% sucrose. Cryopreservation was performed using a two-step freezing protocol to -196 °C with 5% DMSO as a cryoprotectant, following the standard method described by the VKM collection (Redkina and Temraleeva 2025).

### ﻿Microscopy

The morphology of cells and the life cycle of strain VKM Al-436 were investigated using a Leica DM750 light microscope (Germany). The results were captured using a Leica Flexacam C3 color digital camera (Germany). The observation lasted from 1 to 12 months. The important diagnostic characteristics used for identifying the genus *Klebsormidium* include habit, width and length of filaments, cell shape, chloroplast shape, the relative length of chloroplasts to cell length, pyrenoid shape, presence/absence of H-pieces, biseriate filaments, etc. (Rindi et al. 2008). To examine the secretion of slime in the strain, live filaments were stained with India ink to visualize the secreted extracellular mucilage. Morphometric measurements were performed using the Leica Application Suite X software package. For size comparison, we measured 100 exponentially growing cells from the studied strain. The culture was up to 10 weeks old. Dedicated articles were used for morphological identification (Novis 2006; Rindi et al. 2008, 2011; Mikhailyuk et al. 2015; Samolov et al. 2019). The algal taxonomy, which has been accepted in the international electronic database AlgaeBase (Guiry and Guiry 2025) served as the basis for this study.

### ﻿DNA isolation, amplification, purification, and sequencing

The total DNA from the strain VKM Al-436 was isolated using the DNeasy Plant Mini Kit (Qiagen, USA), following the manufacturer’s protocol. A readymade Screen Mix-HS mixture (Eurogen, Russia) was used for amplification. Conditions and primers for the amplification of *rbc*L were described by Skaloud and Rindi (2013) (primers rbcL-KF2 5′-ACTTACTACACTCCTGATTATGA-3′, rbcL-KR2 5′-GGTTGCCTTCGCGAGCTA-3′). The amplification of the *rbc*L gene began with an initial denaturation at 95 °C for 2 min; followed by 40 cycles of denaturing at 94 °C for 1 min, annealing at 47 °C for 1 min, and elongation at 72 °C for 3 min, with a final extension at 72 °C for 8 min). The ITS2 was amplified using primers ITS–AF 5′-CGTTTCCGTAGGTGAACCTGC-3′ and ITS–BR 5′-CATATGCTTAAGTTCAGCGGG-3′. The amplification began with an initial denaturation at 95 °C for 3 min, followed by 35 cycles of denaturing at 95 °C for 30 s, annealing at 57.6 °C for 30 s, and elongation at 72 °C for 1 min, with a final extension at 72 °C for 10 min (Johnson et al. 2007). Target PCR products were detected by electrophoresis on a 1% agarose gel and purified using the Cleanup Mini kit (Evrogen, Russia). The purified amplicons were sequenced using the same primers by the commercial provider Evrogen. The *rbc*L and ITS2 sequences generated in this study were deposited in NCBI GenBank (https://www.ncbi.nlm.nih.gov/genbank) under accession numbers OR838744 and PV368342, respectively.

### ﻿Phylogenetic analysis

The сlosest homologs for the *rbc*L and concatenated ITS2-*rbc*L datasets were identified using the BLASTn algorithm in the NCBI GenBank (Sayers et al. 2022). All sequences met stringent quality criteria, containing no degenerate or unknown nucleotides, with minimum length of 670 bp for *rbc*L and 219 bp for ITS2. The datasets comprised sequences of authentic strains, supplemented with non-authentic, uncultured, environmental sequences showing ≥ 95% similarity to target sequences. Taxonomic nomenclature follows the international electronic database AlgaeBase (Guiry and Guiry 2025). Multiple sequence alignments were generated automatically with the ClustalW algorithm using BioEdit v.7.2.5 (Hall 1999). The optimal nucleotide substitution model for each marker was selected by minimizing the Bayesian information criterion (BIC). Maximum likelihood (ML) phylogenetic analysis was conducted in IQ-TREE 2.2 (Minh et al. 2020). Branch support was assessed with 10,000 replicates of the UFboot2 bootstrap test (Hoang et al. 2018) and with 1,000 replicates of the SH-aLRT test (Shimodaira 2002), the latter of which was performed using the “Building Phylogenetic Tree” module in UGENE. For Bayesian inference (BI), phylogenetic reconstruction was performed using BEAST2 v2.7.5 (Bouckaert et al. 2019) following the recommendations of Barido-Sottani et al. (2018). Six independent Markov Chain Monte Carlo (MCMC) runs were conducted, each with 50 billion generations and sampling every 10,000 steps. The convergence of runs and sufficient sampling were verified in Tracer v1.7 (Rambaut et al. 2018), ensuring all parameters reached an effective sample size (ESS) > 200. The results of the independent runs were combined using LogCombiner v2.7. A maximum clade credibility (MCC) tree was then generated from the combined posterior tree distribution using TreeAnnotator v2.7, with the first 25% of trees discarded as burn-in (Drummond and Bouckaert 2015). Genetic differences between nucleotide sequences were characterized using genetic distances (using the Kimura 2-parameter model), which were calculated in the MEGA 11.0 program (Tamura et al. 2021).

### ﻿Species delimitation

Using a population genetic theory approach, species delimitation involves calculating the ratio K (average pairwise distance between putative species-level clades) to Theta (genetic diversity estimate), performed using the package “KoT-K over Theta” ver. 1 (Spöri et al. 2021). The method LocMin is based on the concept of a ‘dashed discontinuity’ and formalized in a ‘local minimum’ function. This algorithm was implemented using the ‘spider’ package (Brown et al. 2005; Karabanov et al. 2023). Another distance-based method was Assemble Species by Automatic Partitioning (ASAP) (Puillandre et al. 2021), implemented using the ‘asapy’ script from the iTaxoTools package ver. 0.1 (Vences et al. 2021). The distance matrix was calculated using the MEGA 11.0 program (Tamura et al. 2021). Of all the proposed delimitation options, the one with the highest ASAP-score was selected. Also, the generalized mixed Yule coalescent (GMYC) method was used. An ultrametric tree used for this method was reconstructed using the BEAST v. 2.6.2 program (Karabanov et al. 2023; Krivina et al. 2024a, b). The maximum likelihood phylogeny, inferred with IQ-TREE, served as the input for species delimitation using the Poisson Tree Processes (PTP) model with both mlPTP and bPTP algorithms (Zhang et al. 2013). Phylogenetic tree construction was performed using the IQ-TREE software v. 2.2 (Minh et al. 2020) via the phylogenetic tree building block in uGENE. The resulting tree was then converted to NEXUS format and submitted for analysis on the https://species.h-its.org/ web server. The delimitation results (called molecular operational taxonomic units, MOTUs) obtained for each analysis were compared with taxonomically recognized species and classified into the following categories proposed by Magoga et al. (2021): match – all the sequences of the same morphological species were delimited as belonging to the same unit; split – the sequences of a species were delimited as belonging to two or more units; merge – the sequences of two or more species were included in the same unit; and mixture – some sequences of a species were split while others are merged. The group G in which species diversity was best defined by Mikhailyuk et al. (2015) and Samolov et al. (2019) was chosen as a benchmark for evaluating the effectiveness of delimitation methods.

### ﻿Fatty acid analysis

For the fatty acid (FA) analysis of the total lipids (TL), the strain VKM Al-436 was grown in a thermostatically controlled laboratory system for intensive cultivation (Gabrielyan et al. 2023). The cultivation was carried out in 250 ml of BG-11 medium at 27 °C under illumination of 110 μmol photons m^−2^ s^−1^ provided by warm white LEDs from both sides, and with aeration by sterile air enriched with 1.5–2% CO_2_. On the fourth day, the samples from the resulting exponentially growing culture (7–8 mg dry mass) were collected by centrifugation and used for further analysis. FA analysis was performed as described previously (Krivina et al. 2024 b). Briefly, heptadecanoic acid was added to each sample as an internal standard and the samples were hydrolyzed in 1M KOH solution in 80% ethanol, unsaponifiable compounds were removed with n-hexane and the pH of the obtained solution was adjusted to weakly acidic. Then free FAs were extracted by n-hexane and converted into methyl esters (FAMEs). The FA profile of the cell lipids was determined by gas chromatography-mass spectrometry (GC-MS) of the FAMEs, and total lipid content was calculated as the amount of esterified total FAs per gram of biomass dry weight (g d.w.). Three biological replicates, each with two technical replicates, were used for the analysis.

### ﻿Abbreviations

The following abbreviations are used in this manuscript:

**ASAP** Assemble Species by Automatic Partitioning;

**BI** Bayesian inference;

**BP** bootstrap percentage;

**bPTP** Poisson Tree Process with Bayesian inference;

**CBC** compensatory base change;

**FA** fatty acid;

**GMYC** generalized mixed Yule-coalescent;

**ITS** internal transcribed spacer;

**KoT** K over Theta;

**locMin** “Local Minima” function;

**mlPTP** Poisson Tree Process with Maximum Likelihood;

**MOTU** molecular operational taxonomic unit;

**PCR** polymerase chain reaction;

**PP** posterior probability;

**rbcL** ribulose-1,5-bisphosphate carboxylase;

**SAG**The Culture Collection of Algae at Göttingen University;

**SH-aLRT** Shimodaira-Hasegawa-like approximate likelihood ratio test;

**TL** total lipids;

**VKM**The All-Russian Collection of Microorganisms.

## ﻿Results

### ﻿Morphological observations by light microscopy

**Figure 1.**
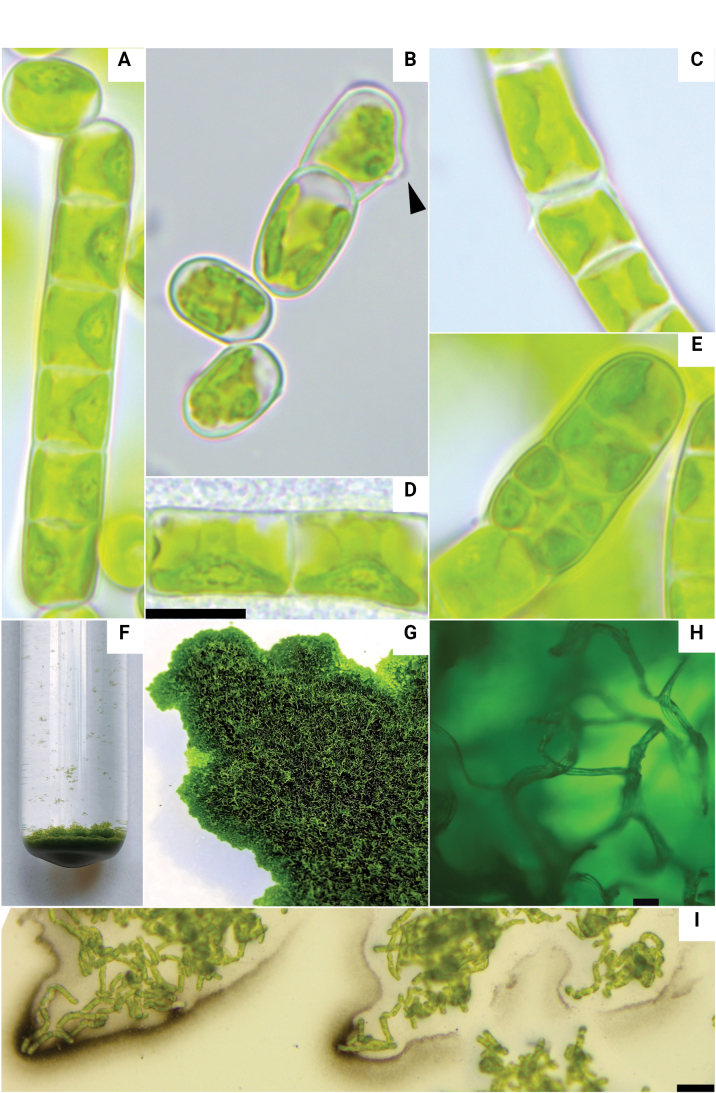
Morphology of strain *Klebsormidium* sp. VKM Al-436. A. General view of filaments and solitary cells; B. Cell with “papilla” (marked with arrow); C. H-pieces of cell wall; D. Cells with clearly visible nucleus and nucleolus; E. Formation of biseriate filaments; F. Flake-like colonies in liquid culture; G. Felt-like colonies on agar plate; H. Clusters of air filaments on agar plate; I. Clusters of filaments stained with India ink. Scale bars: 10 μm (A–E), 50 μm (H–I).

The investigated strain, *Klebsormidium* sp. VKM Al-436, was a filamentous alga that produced filaments of medium (11–50 cells) or long (greater than 50 cells, frequently exceeding 100 cells) length. The filaments were often curved, and at the bends, blunt outgrowths sometimes formed that extended at a certain angle, resembling the initial stage of the formation of lateral branches. It easily disintegrated into short fragments, which sometimes even became unicellular (Fig. 1A). Young cultures had mostly cylindrical or barrel-shaped cells with moderately thickened cell walls and slightly visible or prominent constrictions near cross walls. Cell sizes were (5.7)7.1–18.6(24) × 7.2–10.5 µm with a length to width ratio of 0.6–3. The old cultures of this strain looked completely different. Cells were elongated, barrel- or bean-like, often curved or asymmetrical. The strain had a parietal, girdle-shaped chloroplast with a wavy or smooth margin, which occupied more than half of the inner cell surface (Fig. 1B). The pyrenoid was spherical, small and compact, surrounded by several starch grains (Fig. 1A). H-shaped pieces of the cell wall were sometimes registered (Fig. 1C). The nucleus was clearly visible and was situated opposite to the pyrenoid (Fig. 1D). Occasional presence of biseriate parts of filaments has been observed (Fig. 1E). Vegetative reproduction was achieved through the division of vegetative cells in a single plane followed by the fragmentation of filaments into individual cells or a few-celled fragments (Fig. 1A, C). Some cells differ from others in the formation of “papilla” (Fig. 1B), similar to those of reproductive cells described by Lokhorst (1996). Despite repeated attempts to stimulate zoosporogenesis, we did not observe the release of zoospores or the germination of aplanospores nor the formation of akinetes. Strain usually formed flake-like colonies in liquid culture (Fig. 1F) and felt-like colonies with clusters of air filaments on agar plate (Fig. 1G, H). Clusters of filaments in an axenic culture on an agar plate are surrounded by homogeneous mucilage (Fig. 1I).

### ﻿Phylogenetic analysis

**Figure 2.**
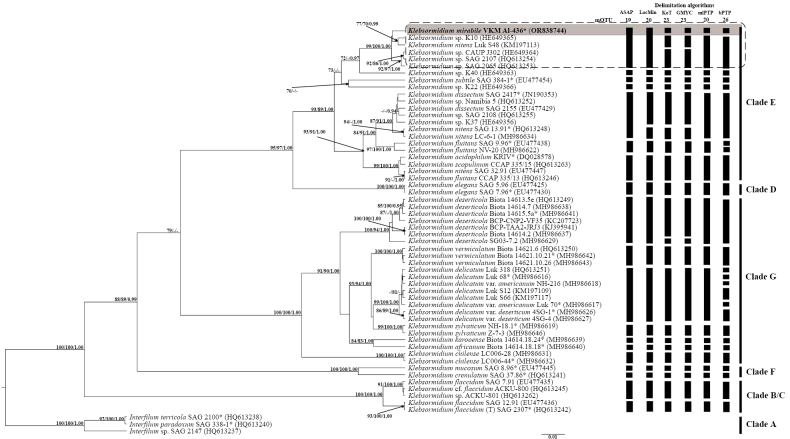
A rooted ultrametric Bayesian phylogenetic tree of the genus *Klebsormidium*, based on the gene *rbc*L sequences (1255 bp). Note. Branch support is indicated as follows: SH-like approximate likelihood ratio test (SH-aLRT) values, bootstrap percentages (BP), and Bayesian posterior probabilities (PP). Values below the following thresholds are omitted: SH-aLRT < 70%, BP < 70%, and PP < 0.9. The nucleotide substitution model used was TIM2+F+I+G4. The studied strain, VKM Al-436, is highlighted in bold. An asterisk (*) denotes authentic strains, and (T) indicates the type species. Black rectangles highlight MOTUs consistently identified by multiple species delimitation methods (ASAP, LocMin, KoT, GMYC, mlPTP, bPTP). The grey rectangle encloses the authentic strain of the new species *K. mirabile* sp. nov. The potential boundaries of this new species are indicated by a dotted line.

In the *rbc*L tree, the studied strain, VKM Al-436, formed an independent cluster together with a number of non-authentic strains of *Klebsormidium* spp. (SH-aLRT – 99%, PP – 100%, BP – 1.00) (Fig. 2). The genetic distances between our strain and other sister strains varied within the range of 1–2% (Suppl. material 1: table S1). Genetic differences between this cluster and strain *Klebsormidium* sp. K40 were 3–4.5%. The genetic distance between the studied strain VKM Al-436 and the closest authentic strain *K. subtile* (Kützing) Mikhailyuk, K.Glaser, A.Holzinger & U.Karsten SAG 384-1 was 3.7%.

**Figure 3.**
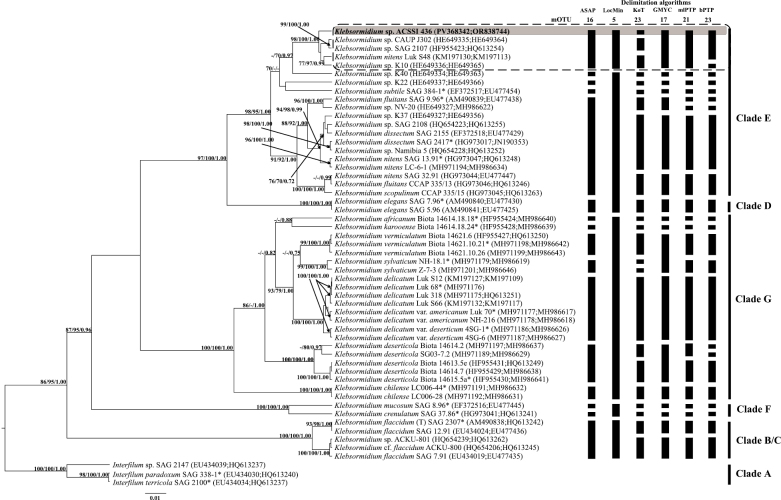
A rooted ultrametric phylogenetic tree of green microalgae from the genus *Klebsormidium*, constructed by the Bayesian inference (BI), based on the ITS2-*rbc*L sequences (1490 bp). Note. Branch support is indicated as follows: SH-like approximate likelihood ratio test (SH-aLRT) values, bootstrap percentages (BP), and Bayesian posterior probabilities (PP). Values below the following thresholds are omitted: SH-aLRT < 70%, BP < 70%, and PP < 0.9. The nucleotide substitution model used was TIM2+F+I+G4. The studied strain, VKM Al-436, is highlighted in bold. An asterisk (*) denotes authentic strains, and (T) indicates the type species. Black rectangles highlight MOTUs consistently identified by multiple species delimitation methods (ASAP, LocMin, KoT, GMYC, mlPTP, bPTP). The grey rectangle encloses the authentic strain of the new species *K. mirabile* sp. nov. The potential boundaries of this new species are indicated by a dotted line.

The tree topology reconstructed from the concatenated ITS2–*rbc*L dataset was largely congruent with that based on the *rbc*L gene alone. The strain VKM Al-436, along with several non-authentic *Klebsormidium* strains, formed a distinct cluster within clade E, with strong statistical support (SH-aLRT – 98%, PP – 100%, BP – 1.00) (Fig. 3). The genetic distances between strain VKM Al-436 and other sister strains were 0.8–1.3% (Suppl. material 2: table S2), distances between this cluster and strain *Klebsormidium* sp. K40 were 2.7–3.2%. The genetic distance between the strain VKM Al-436 and its closest authentic strain *K. subtile*SAG 384-1 was 4.1%.

### ﻿Species delimitation

Different methods of delimitation suggested different numbers of mOTUs (presumable biological species): from 19 (ASAP) to 26 (bPTP) (Fig. 2). The results of the ASAP (ASAP-score = 2.50, P-val = 0.00951, W = 0.000298, Td = 0.0176, p<0.05), LocMin (Td = 0.0154) and mlPTP algorithms were closest to the modern species concept of the genus *Klebsormidium* by reference group G (match proportion – 100%) (Suppl. material 3: fig. S1). The accuracy of the GMYC (P-val = 0.01293047, Tt = 0.004486387, p<0.05), KoT (K = 6) and bPTP algorithms was lower than that of the algorithms described above (match – 86%, split – 14%). The results of all the algorithms showed that our strain was an independent species, distinct from the authentic strain *K. subtile*SAG 384-1.

The number of MOTUs identified using the concatenated ITS2-*rbc*L fragment varied significantly across different delimitation methods, ranging from 5 (LocMin) to 23 (KoT, bPTP) (Fig. 3). ASAP yielded results most consistent with the modern species concept for *Klebsormidium* (reference group G match proportion = 100%, ASAP-score = 6.50, P-val = 0.0141, W = 0.000289, Td = 0.0217, p<0.05) (Suppl. material 4: fig. S2). The accuracy of the mlPTP and bPTP was lower (match – 86%, split – 14%). KoT algorithm demonstrated a tendency to oversplit species (match – 71%, split – 29%). The GMYC algorithm showed the opposite pattern, merging closely related species (match – 71%, merge – 29%). The results of LocMin were the least clear because this algorithm combined all clade G species (match – 0%, merge – 100%).

### ﻿Fatty acid composition.

The FA composition of the TL of the strain VKM Al-436 is presented in Table 1. The TL content was of 109±8 mg/g of d.w. The strain contained 21 distinct FAs. The main fatty acids (>5%) were linoleic (18:2^Δ9,12^), α-linolenic (18:3^Δ9,12,15^), palmitic (16:0), and Δ7,10,13-hexadecatrienoic (16:3^Δ7,10,13^). There were also notable amounts (1–5%) of Δ7,10-hexadecadienoic (16:2^Δ7,10^), stearic (18:0), oleic (18:1^Δ9^), γ-linolenic (18:3^Δ6,9,12^), and arachidonic (20:4^Δ5,8,11,14^) acids. The total amount of polyunsaturated FAs was 72.8±0.6%. The unsaturation index was 1.865±0.023.

**Table 1.** Fatty acid composition of the total lipids of the strain VKM Al-436. The values are the means of three biological replicates, each with two technical replicates, ± standard deviations. Trace amounts (<0.5%) of the following FAs were also detected: 14:0, 16:1^Δ7^, 16:1^Δ9^, 18:1^Δ11^, 20:0, 20:1^Δ11^, 20:3^Δ5,11,14^, 20:3^Δ8,11,14^, 20:3^Δ11,14,17^, 24:0.

FAs	mass %
16:0	18.9±0.3
16:2^Δ7,10^	1.6±0.2
16:3^Δ7,10,13^	5.9±0.2
18:0	1.9±0.1
18:1^Δ9^	3.6±0.4
18:2^Δ9,12^	35.9±1.3
18:3^Δ6,9,12^	1.8±0.0
18:3^Δ9,12,15^	24.5±1.5
20:2^Δ11,14^	0.8±0.1
20:4^Δ5,8,11,14^	1.4±0.1
22:0	0.6±0.1

## ﻿Discussion

Clade E includes a diverse range of species, with varying filament widths from 3–4 μm to 10 μm. The filaments may be strong or fragile, and H-pieces can be absent or present. At the same time, some traits may vary within a single strain, depending on the age and culture conditions. For instance, constrictions at cross walls may occur with aging or under unfavorable conditions. Some features are often not explicitly mentioned when describing species, such as the presence of mucus or the characteristics of macrocolonies. Out of the entire clade E, strain VKM Al-436 is the most similar to *K. dissectum* (F.Gay) H.Ettl & G.Gärtner (Lokhorst 1996) and *K. fluitans* (F.Gay) Lokhorst (Rindi et al. 2011) in terms of filament width, which has rather limited variability. For these species the formation of H-shaped wall remnants is typical (Lokhorst 1996). The tendency towards filament fragmentation makes our strain similar to *K. dissectum*. At the same time, the presence of cells with protruding papilla, which may be reproductive cells, as described by Lokhorst (1996), gives our strain similarity to *K. fluitans*. The species *K. nitens* (Kützing) Lokhorst and *K. subtile*, belonging to the same clade E, differ in filament width (difference 5–7 µm). In addition, *K. nitens* is characterized by the absence of H-pieces. *K. acidophilum* Novis does not exhibit H-shaped wall structures, and its filaments are not easily fragmented. Although in some species, including our proposed new species, the production of zoospores has never been documented (Lokhorst 1996; Samolov et al. 2019), the question of whether the absence of zoosporogenesis can be used to distinguish between species remains unanswered. Shorter and thinner filaments (up to 10.5 μm width in our strain by comparison up to 15 μm width in *K. elegans* Lokhorst), a tendency towards fragmentation, the lack of obvious lobes in the chloroplast and the type of reproduction (zoospores and aplanospores have been observed in *K. elegans*) are the features that differentiate our strain from *K. elegans*, which forms the related clade D. Its main difference from representatives of the clade G is the chloroplast shape, since algae included in this clade have 4 lobes. The strain VKM Al-436 differs from representatives of the clade F in several features: *K. mucosum* (J.B.Petersen) Lokhorst and *K. crenulatum* (Kützing) Lokhorst are characterized by wider, sometimes up to 18–23 μm in width, filaments that are not prone to fragmentation, and cell walls that significantly thicken with culture age. The studied strain differs from *K. flaccidum* (Kützing) P.C.Silva, Mattox & W.H.Blackwell (clade B/C) by having shorter and wider filaments, a pronounced tendency towards fragmentation, and the presence of H-pieces. In addition, zoosporogenesis has been indicated as a type of reproduction for *K. flaccidum*, whereas this type of reproduction is not typical of VKM Al-436.

Conducting a reliable phylogenetic analysis of representatives of the genus *Klebsormidium* can be challenging. Due to the fact that the 18S rRNA gene and internal transcribed spacers (ITS1 and ITS2) are highly conserved molecular markers in this group, their use does not allow for a clear distinction between a number of species, especially within the clade E (Mikhailyuk et al. 2015; Samolov et al. 2019). To accurately determine the phylogenetic position of strain VKM Al-436 within the genus *Klebsormidium*, we conducted analyses using both the variable plastid gene *rbc*L and a concatenated ITS2-*rbc*L fragment. In both reconstructions, the studied strain consistently clustered within clade E, but formed a distinct lineage separate from all authentic strains of the previously described species. The observed genetic distances between VKM Al-436 and its sister strains (1–2% for *rbc*L, 0.8–1.3% for ITS2– *rbc*L) present a taxonomic challenge, as they fall within the ambiguous zone between intraspecific or interspecific variation. On the one hand, in clades F and G, where a taxonomic revision has previously been carried out (Mikhailyuk et al. 2015; Samolov et al. 2019), the minimum interspecific distances are at least 2% for *rbc*L and 3.5% for ITS2– *rbc*L. For example, genetic distances between species *K. mucosum* and *K. crenulatum* were 2% for *rbc*L and 3.9% for ITS2– *rbc*L, *K. karooense* Mikhailyuk and *K. africanum* Mikhailyuk – 2.2% for *rbc*L and 3.5% for ITS2– *rbc*L (Suppl. material 1: table S1, Suppl. material 2: table S2). On the other hand, in the taxonomically complex group E, whose true species richness still needs to be clarified (Kaplan et al. 2012; Mikhailyuk et al. 2015; Samolov et al. 2019), the genetic distances between authentic strains *K. nitens*SAG 13.91 and *K. dissectum*SAG 2417 were only 0.7% for *rbc*L and 1% for ITS2– *rbc*L. Based on the *rbc*L analysis, Kaplan et al. (2012) suggested that these strains belong to the same species. However, using an integrative approach, Mikhailyuk et al. (2015) still attributed them to two different species. Thus, it is difficult to establish clear boundaries of a potentially new species, which is represented by the studied strain VKM Al-436, using the genetic distances of the *rbc*L or ITS2– *rbc*L. At the same time, there is no doubt that the genetic distances between strain VKM Al-436 and the sister authentic strain *K. subtile*SAG 384-1 correspond to the interspecific level (3.7% for *rbc*L, 4.1% for ITS2– *rbc*L).

Currently, the cyber taxonomic approach to determining the taxonomic status of an organism under study is gradually gaining popularity, involving the use of various machine algorithms to distinguish species and reveal the true species richness in the group under investigation (Zhang et al. 2013; Malavasi et al. 2016; Zou et al. 2016; Krivina et al. 2022; Temraleeva and Bukin 2022; Krivina et al. 2024b). This study presents the first comprehensive analysis of species richness in *Klebsormidium* using six delimitation methods applied to the *rbc*L gene and concatenated ITS2–*rbc*L data set: threshold-based (ASAP, LocMin), population genetic (KoT), tree-based (GMYC, mlPTP, bPTP). For *rbc*L, ASAP, LocMin and mlPTP best reflected species diversity in reference clade G, while ASAP showed highest accuracy with ITS2-*rbc*L, followed by mlPTP/bPTP. These preliminary results reflect current limitations in species sampling and persistent discrepancies in species boundaries, particularly in taxonomically complex clade E (Mikhailyuk et al. 2015; Samolov et al. 2019). All methods consistently supported the distinct species status of strain VKM Al-436 from *K. subtile* and other clade E members, with genetic distances corroborating ASAP’s delimitation. Some questions are raised about the boundaries of our new species. The results of the analysis of genetic distances confirm the separation proposed by the ASAP for *rbc*L and the concatenated ITS2–*rbc*L fragment. Thus, among the algorithms evaluated, ASAP proved to be the most effective overall. It outperformed other methods across key parameters, including computational speed, ease of input data preparation, and predictive accuracy. In this regard, it can be assumed that all the components of the cluster, including the strain under study, also belong to this new species. However, detailed morphological studies and additional molecular data of the other strains are needed for confirmation. We note that while MOTUs are pragmatically defined groups of individuals based on similarity, they may not always correspond to biological species (Floyd et al. 2002; Blaxter et al. 2005).

Moreover, to establish a robust phylogenetic framework and find key apomorphic features of streptophyte algae, including *Klebsormidium*, phylogenomic analyses were undertaken. Some molecular signatures were noted, such as the lack of an intron in the *ndh*D gene for strains of *K. crenulatum* and *K. mucosum* (clade F), while this intron is present in other clades of *Klebsormidium* (Glass et al. 2023). However, no specific changes to the taxonomy of the genus were proposed, although there is discussion about the lack of monophyly in *Klebsormidium* (Bierenbroodspot et al. 2024).

Representatives of the clade E of the genus *Klebsormidium* have a very wide geographical distribution. For example, these microalgae were found in Germany, the United Kingdom, the Czech Republic, Denmark, Austria, France, the Netherlands, New Zealand, Chile and even in Namibia. At the same time, among them there are inhabitants of soils, biofilms on various substrates (concrete, roof tiles, moss, artificial stones, snow), as well as various water reservoirs (Novis 2006; Rindi et al. 2008, 2011; Mikhailyuk et al. 2015; Samolov et al. 2019). Based on the above, we can see that the studied strain VKM Al-436 has a fairly typical habitat, since it was isolated from soil of Uzbekistan. Sister strains from the Czech Republic, Germany, and Denmark were isolated from diverse substrates. These included anthropogenic materials like roof tiles and concrete fountain walls, as well as natural substrates such as sandstone, sandy soil, and moss vegetation.

Analyzing the FA composition is essential for assessing the biotechnological potential of microalgal strains. In addition, the fatty acid profile might be employed as an extra chemotaxonomic marker (Dijkman and Kromkamp 2006; Lang et al. 2011; Taipale et al. 2013; Krivina et al. 2024b). The initial step in developing the chemotaxonomy of the group under study is to thoroughly examine the FA profile of the strains that were correctly identified. Unfortunately, research on the FA composition in the genus *Klebsormidium* remains insufficient to identify chemotaxonomic markers at the genus and species level. Nevertheless, it can be noted that palmitic, linoleic, and α-linolenic acids as major FAs were found in most of the studied *Klebsormidium* strains (Lang et al. 2011; Buse et al. 2013; Liu et al. 2016; Qiu et al. 2020; Xu et al. 2021; Santunione et al. 2024). Arachidonic acid (ARA) was also found in some strains, identified as *Klebsormidium* sp., *K. flaccidum*, *K. fluitans*, and *K. nitens* (Lang et al. 2011; Buse et al. 2013; Liu et al. 2016; Qiu et al. 2020; Santunione et al. 2024). However, the reference strain *K. nitens*SAG 13.91 did not contain ARA and additionally differs from the studied strain VKM Al-436 in its lack of Δ7,10,13-hexadecatrienoic and γ-linolenic acids (Lang et al. 2011). The reported FA profile of *Klebsormidium* sp. UMACC 227, a strain isolated from a former oil spill site in the Windmill Islands region of Antarctica, differs significantly from that of other *Klebsormidium* strains that have been examined. Under all tested conditions (growth at temperatures of 4–20 °C, under photosynthetically active radiation (PAR) only or under PAR+UVA or PAR+UVB), this strain exhibited a much higher stearic acid content (~up to 50%), significantly lower levels of linoleic and α-linolenic acids, and a complete absence of ARA (Chiew-Yen et al. 2004; Teoh et al. 2004). However, the affiliation of this strain within the genus *Klebsormidium* has not been confirmed by phylogenetic analysis.

Some strains of the genus *Klebsormidium* were shown to be biotechnologically interesting due to their stress resistance, high growth rate and ability to accumulate lipids rich in linoleic acid which make them suitable for human and animal nutrition and for commercial production of microalgal oils (Stoyneva-Gärtner et al. 2019; Fang et al. 2022; Segura-Morales et al. 2024). The origin of the studied strain VKM Al-436 from soil and its high content of linoleic acid make this strain promising for the further study of its potential in biotechnology.

## ﻿Taxonomic conclusions

### ﻿ Klebsormidium mirabile

Taxon classificationPlantaeKlebsormidialesKlebsormidiaceae

Tukhtaboeva, Krivina, Redkina & Temraleeva sp. nov.

6F0723F0-49D5-5A7F-8944-ED3C86148F34

#### Description.

Filaments predominantly uniseriate and unbranched, curved, with a medium or long length (over 100 cells), easily disintegrated into short fragments (sometimes unicellular) and forming flake-like colonies in liquid culture and felt-like colonies with clusters of air filaments on agar plate. In young cultures cells mostly cylindrical or barrel-shaped with moderately thickened cell walls (sometimes with H-pieces) and slightly visible or prominent constrictions near the cross walls. Cell sizes (5.7)7.1–18.6(24)×7.2–10.5 µm with a length to width ratio of 0.6–3. In old cultures cells elongated, barrel- or bean-like, often curved or asymmetrical. Chloroplast single and parietal, girdle-shaped with a wavy or smooth margin, which occupied more than one half of cell periphery. Pyrenoid single, spherical, small and compact, surrounded by several distinct starch grains. Nucleus clearly visible and situated opposite to the pyrenoid. Mucilage present. Vegetative multiplication by fragmentation of filaments into unicellular or few-cellular fragments. Zoospores, aplanospores, gametes, akinetes not observed.

#### Holotype.

Cryopreserved material in a metabolically inactive state from authentic culture VKM Al-436 stored under designation VKM Al-436cryo in the All-Russian Collection of Microorganisms (VKM) at the Skryabin Institute of Biochemistry and Physiology of Microorganisms at the Pushchino Biological Research Center of the Russian Academy of Sciences, Pushchino, Russia.

#### Authentic culture.

Culture of authentic strain VKM Al-436 was kept at the All-Russian Collection of Microorganisms (VKM) at the Skryabin Institute of Biochemistry and Physiology of Microorganisms at the Pushchino Biological Research Center of the Russian Academy of Sciences, Pushchino, Russia.

#### Representative illustration.

Fig. 1A–H.

#### Molecular analyses.

Phylotype (OR838744) differs from other species of genus *Klebsormidium* in *rbc*L phylogeny.

#### Habitat.

Soil.

#### Type locality.

Uzbekistan, the Chust district of the Namangan region, from soil of the Chust-Pop Hills in the Fergana Valley, 40°52'37.14"N, 70°59'44.76"E.

#### Etymology.

The specific epithet is coined for the wonderful appearance of our new species.

## Supplementary Material

XML Treatment for Klebsormidium mirabile

## ﻿Additional information

### Use of AI

The authors declare that no generative AI was used in the creation of this manuscript.

### Author ORCIDs

Yulduzkhon Tukhtaboeva https://orcid.org/0009-0008-2209-3582

Elena Krivina https://orcid.org/0000-0002-0849-5832

Vera Redkina https://orcid.org/0000-0002-4908-5098

Aleksey Portnov https://orcid.org/0000-0002-3749-4265

Maria Sinetova https://orcid.org/0000-0001-9931-1762

Anna Temraleeva https://orcid.org/0000-0002-3445-0507

### Data availability

All authors confirm that the data supporting the findings of this study are available within the article and its supplementary material. In addition, raw data sup-porting this study’s findings are available from the corresponding author upon a reasonable request.

## Supplementary materials

Supplementary material 1 *rbc*L genetic differences between *Klebsormidium mirabile* sp. nov. VKM Al-436 and other members of the genus *Klebsormidium* in %. Data type doc https://binary.pensoft.net/file/1460479This dataset is made available under the Open Database License (http://opendatacommons.org/licenses/odbl/1.0/). The Open Database License (ODbL) is a license agreement intended to allow users to freely share, modify, and use this Dataset while maintaining this same freedom for others, provided that the original source and author(s) are credited.Yulduzkhon Tukhtaboeva, Elena Krivina, Vera Redkina, Aleksey Portnov, Maria Sinetova, Anna Temraleeva

Supplementary material 2 ITS2–*rbc*L genetic differences between *Klebsormidium mirabile* sp. nov. VKM Al-436 and other members of the genus *Klebsormidium* in %. Data type doc https://binary.pensoft.net/file/1460480This dataset is made available under the Open Database License (http://opendatacommons.org/licenses/odbl/1.0/). The Open Database License (ODbL) is a license agreement intended to allow users to freely share, modify, and use this Dataset while maintaining this same freedom for others, provided that the original source and author(s) are credited.Yulduzkhon Tukhtaboeva, Elena Krivina, Vera Redkina, Aleksey Portnov, Maria Sinetova, Anna Temraleeva

Supplementary material 3 Evaluation of the effectiveness of different delimitation methods using rbcL for the genus *Klebsormidium* Data type doc https://binary.pensoft.net/file/1460481This dataset is made available under the Open Database License (http://opendatacommons.org/licenses/odbl/1.0/). The Open Database License (ODbL) is a license agreement intended to allow users to freely share, modify, and use this Dataset while maintaining this same freedom for others, provided that the original source and author(s) are credited.Yulduzkhon Tukhtaboeva, Elena Krivina, Vera Redkina, Aleksey Portnov, Maria Sinetova, Anna Temraleeva

Supplementary material 4 Evaluation of the effectiveness of different delimitation methods using the ITS2–*rbc*L fragment for the genus *Klebsormidium* Data type doc https://binary.pensoft.net/file/1460482This dataset is made available under the Open Database License (http://opendatacommons.org/licenses/odbl/1.0/). The Open Database License (ODbL) is a license agreement intended to allow users to freely share, modify, and use this Dataset while maintaining this same freedom for others, provided that the original source and author(s) are credited.Yulduzkhon Tukhtaboeva, Elena Krivina, Vera Redkina, Aleksey Portnov, Maria Sinetova, Anna Temraleeva
