# Supplementary material for: ﻿Species delimitation in the genus Klebsormidium (Klebsormidiophyceae, Charophyta), including description of Klebsormidium mirabile sp. nov. with high content of polyunsaturated fatty acids
Source: PhytoKeys. 2025 Nov 7;266:53–74. doi: 10.3897/phytokeys.266.158514 (PMC12679119; doi:10.3897/phytokeys.266.158514)
Supplement: Supplementary material 1 — rbcL genetic differences between Klebsormidium mirabile sp. nov. VKM Al-436 and other members of the genus Klebsormidium in %. [file phytokeys-266-053_article-158514__-s001.doc]

**Supplementary material 1**

**Table S1.** *rbc*L genetic differences between *Klebsormidium mirabile* sp. nov. VKM Al-436 and other members of the genus *Klebsormidium* in %

|  | ***Klebsormidium*  *mirabile* sp. nov. VKM Al-436** | *Klebsormidium* sp.  K10, Luk S48 | *Klebsormidium* sp.  CAUP J302, SAG 2107, SAG 2066 | *Klebsormidium* sp. K40 | *K. subtile* SAG 384-1* | *K. dissectum* SAG 2417* | *K. nitens* SAG 13.91* | *K. fluitans* SAG 9.96* | *K. acidophilum* KRIV* | *K. elegans* | *K. deserticola* | *K. vermiculatum* | *K. delicatum* | *K. sylvaticum* | *K. karooense* | *K. africanum* | *K. chilense* | *K. mucosum* | *K. crenulatum* | *K. flaccidum* SAG 7.91, ACKU-800, ACKU-801 | *K. flaccidum* SAG 2307*, SAG 12.92 |
| --- | --- | --- | --- | --- | --- | --- | --- | --- | --- | --- | --- | --- | --- | --- | --- | --- | --- | --- | --- | --- | --- |
| ***Klebsormidium mirabile* sp. nov. VKM Al-436*** | 0 | - | - | - | - | - | - | - | - | - | - | - | - | - | - | - | - | - | - | - | - |
| *Klebsormidium* sp. K10, Luk S48 | 1-2 | 1.5 | - | - | - | - | - | - | - | - | - | - | - | - | - | - | - | - | - | - | - |
| *Klebsormidium* sp. CAUP J302, SAG 2107, SAG 2066 | 1-1.2 | 1-2.2 | 0.2-0.5 | - | - | - | - | - | - | - | - | - | - | - | - | - | - | - | - | - | - |
| *Klebsormidium* sp. K40 | 3 | 3.5-4.5 | 3.5-3.7 | 0 | - | - | - | - | - | - | - | - | - | - | - | - | - | - | - | - | - |
| *K. subtile* SAG 384-1* | 3.7 | 3.7-4.5 | 4.3-4.5 | 4.2 | 0 | - | - | - | - | - | - | - | - | - | - | - | - | - | - | - | - |
| *K. dissectum* SAG 2417* | 4.5 | 4.5-5.3 | 4-4.3 | 4 | 4.3 | 0 | - | - | - | - | - | - | - | - | - | - | - | - | - | - | - |
| *K. nitens* SAG 13.91* | 4.3 | 4.3-5 | 3.8-4 | 3.7 | 4 | 0.8 | 0 | - | - | - | - | - | - | - | - | - | - | - | - | - | - |
| *K. fluitans* SAG 9.96* | 3.5 | 4-4.8 | 4-4.3 | 3.7 | 4.2 | 2.2 | 2 | 0 | - | - | - | - | - | - | - | - | - | - | - | - | - |
| *K. acidophilum* KRIV* | 3.5 | 3.5-4.3 | 4-4.3 | 3.5 | 4.2 | 2 | 1.7 | 1.7 | 0 | - | - | - | - | - | - | - | - | - | - | - | - |
| *K. elegans* | 5 | 5.3-5.8 | 4.5-4.8 | 5.3 | 5 | 4.8 | 4.5 | 5.5 | 5.8 | 0 | - | - | - | - | - | - | - | - | - | - | - |
| *K. deserticola* | 12.5-12.8 | 12.5-13.8 | 11.9-12.5 | 10.7-11 | 11.6-11.9 | 11.1-11.3 | 10.8-11 | 11.3-11.6 | 11.6-11.9 | 11.6-11.9 | 0-0.5 | - | - | - | - | - | - | - | - | - | - |
| *K. vermiculatum* | 11.9 | 11.9-12.1 | 11.3-11.9 | 10.7 | 12.2 | 10.4 | 10.2 | 10.7 | 11 | 12.2 | 4-4.3 | 0 | - | - | - | - | - | - | - | - | - |
| *K. delicatum* | 12.5-12.8 | 12.5-12.8 | 11.9-12.8 | 11.3-11.6 | 12.8-13.1 | 11.1-11.4 | 11.4-11.7 | 11.3-11.6 | 11.6-11.9 | 12.2-12.5 | 4-4.5 | 2.5-2.7 | 0.2 | - | - | - | - | - | - | - | - |
| *K. sylvaticum* | 13-13.3 | 13-13.6 | 12.7-13.3 | 11.8-12.1 | 13-13.3 | 11.3-11.6 | 11-11.3 | 11.6-11.9 | 11.3-11.6 | 13-13.3 | 5.3-5.6 | 3-3.2 | 3.5-4 | 0.2 | - | - | - | - | - | - | - |
| *K. karooense* | 13.4 | 13.4-13.6 | 12.8-13.4 | 12.2 | 13 | 11.6 | 11.3 | 12.5 | 12.2 | 13.1 | 2.7 | 3.5 | 4-4.3 | 5-5.3 | 0 | - | - | - | - | - | - |
| *K. africanum* | 13.4 | 13.4-14.6 | 12.8-13.1 | 12.5 | 14.3 | 12.2 | 11.9 | 12.5 | 12.8 | 12.5 | 3.5 | 4.3 | 4-4.5 | 5.3-5.6 | 2.2 | 0 | - | - | - | - | - |
| *K. chilense* | 14.5 | 13.9-14.2 | 13-13.6 | 12.7 | 12.4 | 11.8 | 11.5 | 12.4 | 12.7 | 12.4 | 6.1 | 6.9 | 6.6-7.2 | 7.1-7.4 | 5.8 | 6.9 | 0 | - | - | - | - |
| *K. mucosum* | 12.5 | 11.9-12.2 | 11-11.6 | 11.6 | 11.9 | 10.7 | 11 | 11.3 | 11.9 | 11.3 | 14.9-15.2 | 13.6 | 13-13.3 | 15.4-15.7 | 13.9 | 13 | 12.7 | 0 | - | - | - |
| *K. crenulatum* | 13.7 | 13.1-13.3 | 12.2-12.8 | 12.8 | 13.7 | 12.5 | 12.8 | 13 | 13.7 | 13 | 15.5-15.8 | 13.9 | 13.6-13.9 | 16.3-16.6 | 13.9 | 13 | 13.6 | 2 | 0 | - | - |
| *K. flaccidum* SAG 7.91, ACKU-800, ACKU-801 | 13.6-13.9 | 14.5-15.1 | 14.2-14.8 | 15.1-15.4 | 15.7-16 | 14.2-14.5 | 15.2-15.5 | 13.6-13.9 | 15.5-15.8 | 15.5-15.8 | 16.1-16.8 | 15.4-15.8 | 15.2-15.8 | 17-17.6 | 16.1 | 15.5-15.8 | 15.8-16.1 | 13.7-14 | 0.2 | 0 | - |
| *K. flaccidum* SAG 2307*, SAG 12.92 | 13 | 13.9-14.2 | 13.6-13.9 | 14.8 | 15.1 | 13.3 | 14.2 | 13.3 | 14.6 | 14.9 | 15.5-15.8 | 14.8 | 14.6-14.9 | 16.3-16.7 | 16.4 | 14.2 | 15.1 | 13.3 | 2.2 | 2 | 0 |

**^*^**‒ authentic strain.
