# Supplementary material for: ﻿Species delimitation in the genus Klebsormidium (Klebsormidiophyceae, Charophyta), including description of Klebsormidium mirabile sp. nov. with high content of polyunsaturated fatty acids
Source: PhytoKeys. 2025 Nov 7;266:53–74. doi: 10.3897/phytokeys.266.158514 (PMC12679119; doi:10.3897/phytokeys.266.158514)
Supplement: Supplementary material 2 — ITS2–rbcL genetic differences between Klebsormidium mirabile sp. nov. VKM Al-436 and other members of the genus Klebsormidium in %. [file phytokeys-266-053_article-158514__-s002.doc]

**Supplementary material 3**

**Table S2.** ITS2‒*rbc*L genetic differences between *Klebsormidium mirabile* sp. nov. VKM Al-436 and other members of the genus *Klebsormidium* in %

|  | ***Klebsormidium*  *mirabile* sp. nov. VKM Al-436** | *Klebsormidium* sp. K10, Luk S48 | *Klebsormidium* sp. CAUP J302, SAG 2107 | *Klebsormidium* sp. K40 | *K. subtile* SAG 384-1* | *K. dissectum* SAG 2417* | *K. nitens* SAG 13.91* | *K. fluitans* SAG 9.96* | *K. elegans* | *K. deserticola* | *K. vermiculatum* | *K. delicatum* | *K. sylvaticum* | *K. karooense* | *K. africanum* | *K. chilense* | *K. mucosum* | *K. crenulatum* | *K. flaccidum* SAG 7.91, ACKU-800, ACKU-801 | *K. flaccidum* SAG 2307*, SAG 12.92 |
| --- | --- | --- | --- | --- | --- | --- | --- | --- | --- | --- | --- | --- | --- | --- | --- | --- | --- | --- | --- | --- |
|
| ***Klebsormidium mirabile* sp. nov. VKM Al-436*** | 0 | - | - | - | - | - | - | - | - | - | - | - | - | - | - | - | - | - | - | - |
| *Klebsormidium* sp. K10, Luk S48 | 0.7-1.3 | 0.7 | - | - | - | - | - | - | - | - | - | - | - | - | - | - | - | - | - | - |
| *Klebsormidium* sp. CAUP J302, SAG 2107 | 1 | 0.7-1.4 | 0.2 | - | - | - | - | - | - | - | - | - | - | - | - | - | - | - | - | - |
| *Klebsormidium* sp. K40 | 2.7 | 3.1-3.2 | 3.1-3.2 | 0 | - | - | - | - | - | - | - | - | - | - | - | - | - | - | - | - |
| *K. subtile* SAG 384-1* | 4.1 | 3.6-4.2 | 4-4.2 | 4.2 | 0 | - | - | - | - | - | - | - | - | - | - | - | - | - | - | - |
| *K. dissectum* SAG 2417* | 3.9 | 3.6-4.1 | 3.5 | 3.8 | 4.4 | 0 | - | - | - | - | - | - | - | - | - | - | - | - | - | - |
| *K. nitens* SAG 13.91* | 3.9 | 3.6-4.2 | 3.5-3.6 | 4 | 4.4 | 1 | 0 | - | - | - | - | - | - | - | - | - | - | - | - | - |
| *K. fluitans* SAG 9.96* | 4.2 | 3.8-4.3 | 4 | 4.2 | 4.6 | 2.1 | 2.4 | 0 | - | - | - | - | - | - | - | - | - | - | - | - |
| *K. elegans* | 7.5 | 7.1-7.7 | 6.7-6.8 | 7.1 | 7.1 | 7 | 6.9 | 7.1 | 0 | - | - | - | - | - | - | - | - | - | - | - |
| *K. deserticola* | 11.9-12.4 | 11.6-12.7 | 11.7-12.2 | 11-11.4 | 12.1-12.6 | 11.2-11.8 | 10.9-11.5 | 11.3-12 | 12.4-12.8 | 0-1.5 | - | - | - | - | - | - | - | - | - | - |
| *K. vermiculatum* | 11.3-11.5 | 11.4-11.5 | 11.3-11.7 | 11.3-11.5 | 11.9-12.2 | 10.8-11.1 | 10.8-11 | 10.9-11.3 | 12.2-12.3 | 5-5.5 | 0.1-0.2 | - | - | - | - | - | - | - | - | - |
| *K. delicatum* | 11.9-12.6 | 11.9-12.7 | 11.2-12.4 | 11.1-12.1 | 11.8-12.9 | 10.2-12 | 11-12.1 | 10.5-11.9 | 11.8-12.9 | 5.8-6.5 | 3.3-3.8 | 0-1.1 | - | - | - | - | - | - | - | - |
| *K. sylvaticum* | 11.6-11.7 | 11.3-11.9 | 11.2-11.8 | 11.1-11.6 | 11.7-12.1 | 10.3-11 | 10.8-11 | 10.5-11.2 | 12.2-12.3 | 5.8-6.6 | 2.7-2.9 | 3.5-4.2 | 0.5 | - | - | - | - | - | - | - |
| *K. karooense* | 11.6 | 11.5-11.9 | 11.1-11.5 | 10.8 | 11.2 | 10.1 | 10.6 | 10.8 | 12.4-12.5 | 5-5.5 | 4.1-4.5 | 5-5.7 | 3.9-4.5 | 0 | - | - | - | - | - | - |
| *K. africanum* | 12 | 11.9-12.2 | 11.9-12.1 | 11.8 | 12.7 | 11.8 | 11.7 | 11.8 | 12.8 | 4.8-5.5 | 4.1-4.5 | 3.9-4.6 | 4.5 | 3.5 | 0 | - | - | - | - | - |
| *K. chilense* | 13.7-13.8 | 13.3-13.8 | 12.7-12.9 | 12.6-12.7 | 12.6-12.7 | 12.3 | 11.9 | 11.9-12 | 13 | 8.2-8.7 | 7.3-7.4 | 7.9-8.4 | 7.1 | 7 | 7.5 | 0 | - | - | - | - |
| *K. mucosum* | 11.8 | 11.9-12.1 | 11.4-11.9 | 11.5 | 12 | 11.7 | 12.2 | 11.6 | 13.3-13.4 | 13.1-14.7 | 13.7-14.3 | 11.9-14.4 | 13.1-14.4 | 11.5 | 13.3 | 12.8 | 0 | - | - | - |
| *K. crenulatum* | 11.5 | 11.5-11.6 | 11.3-11.5 | 11.2 | 12 | 11.8 | 11.9 | 12 | 13-13.1 | 13.1-14.4 | 13.2-13.5 | 11.4-13.7 | 13.1-14.2 | 11.2 | 12.6 | 12.6-12.8 | 3.9 | 0 | - | - |
| *K. flaccidum* SAG 7.91, ACKU-800, ACKU-801 | 11.6-12 | 11.5-12.6 | 11.4-11.9 | 12.2-12.7 | 12.6 | 11.6-12.2 | 11.9-12.4 | 11.4-11.9 | 13.3-13.6 | 14.7-16.2 | 14.3-15 | 13-15.3 | 14.7-16.1 | 13.6-14.2 | 14.3-14.7 | 14.9-15.3 | 14.4-14.5 | 14.4-14.6 | 0.1-0.2 | - |
| *K. flaccidum* SAG 2307*, SAG 12.92 | 11.4-11.5 | 11.3-12.2 | 11.3-11.4 | 12.2-12.4 | 12.7-13.1 | 11.2-11.4 | 11.5-11.6 | 11.3 | 13.4-13.5 | 14-15.3 | 13.9-14.2 | 12.3-14.6 | 13.9-15.2 | 12.7-12.9 | 13.6-13.8 | 14-14.3 | 14.1-14.5 | 14.4-14.3 | 1.7-1.8 | 0 |

**^*^**‒ authentic strain.
