# Supplementary material for: ﻿Species delimitation in the genus Klebsormidium (Klebsormidiophyceae, Charophyta), including description of Klebsormidium mirabile sp. nov. with high content of polyunsaturated fatty acids
Source: PhytoKeys. 2025 Nov 7;266:53–74. doi: 10.3897/phytokeys.266.158514 (PMC12679119; doi:10.3897/phytokeys.266.158514)
Supplement: Supplementary material 3 — Evaluation of the effectiveness of different delimitation methods using rbcL for the genus Klebsormidium [file phytokeys-266-053_article-158514__-s003.doc]

**Supplementary material 3**


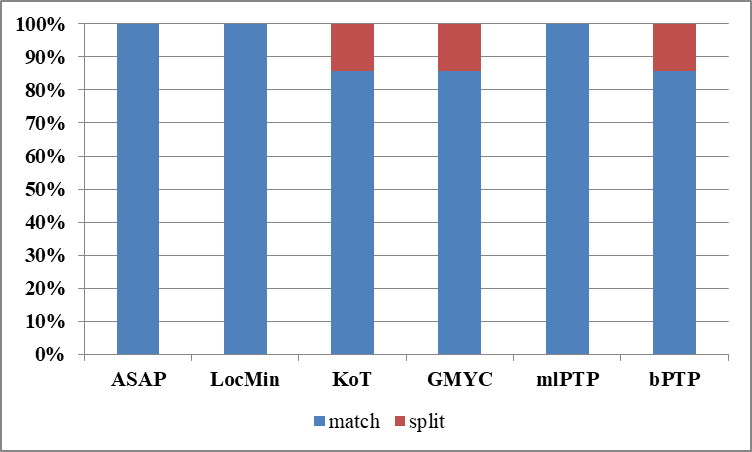


**Figure S1.** Evaluation of the effectiveness of different delimitation methods using *rbc*L for the genus *Klebsormidium*.
