# Supplementary material for: ﻿Species delimitation in the genus Klebsormidium (Klebsormidiophyceae, Charophyta), including description of Klebsormidium mirabile sp. nov. with high content of polyunsaturated fatty acids
Source: PhytoKeys. 2025 Nov 7;266:53–74. doi: 10.3897/phytokeys.266.158514 (PMC12679119; doi:10.3897/phytokeys.266.158514)
Supplement: Supplementary material 4 — Evaluation of the effectiveness of different delimitation methods using the ITS2–rbcL fragment for the genus Klebsormidium [file phytokeys-266-053_article-158514__-s004.doc]

**Supplementary material 4**


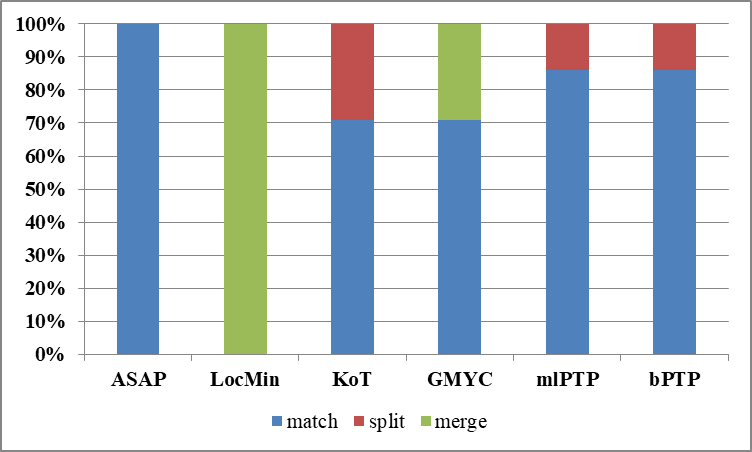


**Figure S2.** Evaluation of the effectiveness of different delimitation methods using the ITS2‒*rbc*L fragment for the genus *Klebsormidium*.
